# Barriers to Early Utilization of Palliative Care in Heart Failure: A Narrative Review

**DOI:** 10.3390/healthcare8010036

**Published:** 2020-02-07

**Authors:** Massimo Romanò

**Affiliations:** Interdepartmenntal University Research Center for Palliative Care, Universityof Milan, Milano 7-20122, Italy; max.romano51@gmail.com; Tel.: +393488131566

**Keywords:** palliative care, supportive care, heart failure, end of life

## Abstract

Palliative care is indicated in patients with heart failure since the early phases of the disease, as suggested by international guidelines. However, patients are referred to palliative care very late. Many barriers could explain the gap between the guidelines’ indications and clinical practice. The term palliative is perceived as a stigma by doctors, patients, and family members because it is charged with negative meanings, a poor prognosis, and no hope for improvement. Many authors prefer the term supportive care, which could facilitate a discussion between doctors, patients, and caregivers. There is substantial variation and overlap in the meanings assigned to these two terms in the literature. Prognosis, as the main indication to palliative care, delays its implementation. It is necessary to modify this paradigm, moving from prognosis to patients’ needs. The lack of access to palliative care programs is often due to a lack of palliative care specialists and this shortage will be greater in the near future. In this study, a new model is proposed to integrate early over the course of the disease the palliative care (PC) specialist in the heart failure team, allowing to overcome the barriers and to achieve truly simultaneous care in the treatment of heart failure (HF) patients.

## 1. Introduction 

The prevalence of heart failure (HF) in Western countries is 2.2%–2.5%, with 5–10 new cases per 1000 people/year [1]. The prevalence increases with age. HF is a major cause of hospitalization in high-income countries and has a negative impact on quality of life.

Patients with HF have the same symptom burden as those with cancer, with the same prevalence of depression and reduced spiritual wellbeing (35%–40% of patients) [2]. Over half of all HF patients complain of dyspnea, pain, asthenia, and thirst. However, in the more advanced stages of HF, the number of symptoms exceeds those associated with advanced cancer, depression is more frequent, and spiritual malaise is more severe [2]. Patients with HF, therefore, have great palliative care (PC) needs and their clinical impact increases significantly in the last six months of life. The World Atlas of PC, published by the WHO in 2014, estimates that in the coming years, slightly over 20 million people in the world will need PC for the management of incurable “life-limiting” or “life-threatening” disease [3].

The guidelines of the main international scientific societies [4,5,6,7] all refer to the need to start end-of-life (EOL)/PC for patients with end-stage HF, to optimize their quality of life by providing adequate symptom control, in accordance with the wishes of patients and their families. The European Society of Cardiology (ESC) guidelines acknowledge that it is difficult to identify which patients require specialist PC or EOL care. The criteria they propose include a high number of hospital admissions over the past year, frequent HF relapses, a NYHA class IV, a progressive decline in the quality of life, and non-eligibility for a heart transplant or mechanical circulatory support [4]. The American Heart Association / American College of Cardiology (AHA-ACC) guidelines recommend initiating PC to improve the quality of life in symptomatic HF patients. Orders for such care should be included in the discharge plan following hospitalization for an acute event (class 1 recommendation, evidence level B) [7].

The same recommendations are present in Canadian Cardiovascular Society guidelines on heart failure.

However, the number of patients who need PC differs markedly from the number who actually receive it, especially in patients with cardiovascular diseases. Only 4% of HF patients in Great Britain receive PC, and this figure has remained constant over the years [8]. In the world of PC in England, 50% of the deaths involved cancer patients, as opposed to only 7% for patients with HF. Moreover, one third of those HF patients had been included in a PC program only during the last week of life [9]. Warraich et al. recently analyzed data from a multicenter registry on over 1800 North American patients referred for PC for cardiovascular disease (HF in 70% of the cases) between 2015 and 2017. Thirty percent of the referrals were for patients with Palliative Performance Scores (PPS) between 0% and 30% (dying or bedridden patients), and only 12% of the patients were referred by cardiologists [10]. In essence, patients with HF are rarely referred for PC, but when they are, the referral comes quite late, at the end of life.

What are the reasons for this gap between the practices advocated by the guidelines and those observed in clinical practice? Differences in how PC is used in various medical fields cannot be attributed to the contents of the European guidelines, which are similar for patients with HF, respiratory failure, or cancer [11]. The problem seems to be more of a cultural one, linked to the belief that PC is destined exclusively for patients dying or suffering from cancer, or to fears that initiating PC means all attempts to actively treat the HF will be discontinued. Furthermore, not all HF patients are being cared for by cardiologists, and this raises the concern that PC might be started on the “wrong” patient, that the non-cardiologist care provider may not have considered all the available options. The problem is further complicated by the objective complexities involved in formulating a short- to mid-term prognosis for patients with HF [4]. The barriers also stem from limited awareness—from patients and doctors alike—that HF is a progressive and potentially fatal disease, that the prognosis is uncertain, and that most physicians are poorly prepared to discuss treatment goals with their patients and colleagues [12,13].

In advanced HF patients, the available therapeutic strategies when PC starts are substantially related to palliative inotropes administration (dobutamine in continuous infusion or levosimendan in intermittent infusion) and to the management of implanted cardiac devices (Implantable cardioverter defibrillators (ICD), cardiac pacemakers (PM), left ventricular assist devices (LVAD)) and their possible deactivation at the end of life [5].

## 2. Stigma of Referring Patients to Palliative Care

One fundamental point to consider is the conceptual overlap between PC and EOL, although the non-synonymous nature of the terms has long been stressed by the WHO [14]. Some authors feel that the term palliative care hinders the early initiation of these measures, even for cancer patients. Patients, family members, and physicians see PC as something that is destined for a patient who has reached the end of life and is without hope and that this care is provided in a hospice program after all attempts to actively treat the disease have been suspended [15].

The same obstacles to early implementation of PC are found in the literature regarding pediatric oncology [16,17]—some surveys of health care professionals showed that the term palliative care is associated with death and dying, which delays the start of PC. The need for education and training to modify this relevant factor is outlined in this study.

The debate over the terminology and the role it plays in preventing physicians from starting PC has led to the proposal of the alternative term supportive care (SC). The Multinational Association of Supportive Care in Cancer (MASCC) defines SC care in cancer as “the prevention and management of the adverse effects of the disease and its treatment. This includes management of physical and psychological symptoms and side effects across the cancer experience through diagnosis, through anticancer treatment, to post-treatment care. Enhancing rehabilitation, secondary cancer prevention, survivorship, and end-of-life care are integral to supportive care” [18]. The term palliative, in contrast, is frequently perceived by oncologists as an obstacle because it destroys hope and causes stress—the term supportive is certainly preferred by patients and their relatives [19].

Oncologists find that it is easier to involve patients with early-stage disease in an SC program than in one providing PC [19]. An analysis of the referrals to a PC service in the United States before and after the name of the service was changed to read “supportive care” found that the change in terminology resulted in a 41% increase in the number of inpatient referrals and earlier referrals for outpatient PC. The authors suggested that the terms be promptly changed [20]. The European Society for Medical Oncology (ESMO) has proposed resolving the debate by using the term patient-centered care, which comprises both of the underlying concepts [21].

The stigma attached to the term palliative is even greater among specialists in non-oncological diseases, who have always been reluctant to discuss prognoses and EOL issues with patients and their families. Pulmonologists and cardiologists in Belgium were recently surveyed on the views and current practices for incorporating palliative measures into the care of patients with chronic obstructive pulmonary disease (COPD)/HF. The term palliative care emerged as having a bad reputation, with unpleasant connotations for patients and family members [22].

Because the prognosis for HF/COPD is difficult to define and neither disease is identified with death in the minds of the general public, the survey participants reportedly avoided even mentioning the existence of PC services, especially in the earlier stages of illness [22]. The term supportive care was preferred, since it was not directly associated with death.

## 3. Terminology as a Barrier to Receive PC

Cardiac surgeons in the United States are also addressing the problem of terminology in relation to cardiac surgery patients admitted in a critical condition to intensive care units. Katz notes that the team responsible for EOL issues in this setting is generally called the PC team, a term he feels is in conflict with the basic objective of cardiac surgery, which is to restore the patient to a life of significant quality outside the hospital. The term palliative sends a negative message, while the term supportive is considered more appropriate and more likely to lead the surgeon to consult the team [23]. In an editorial comment, Nakagawa emphasized that the difficulties with the term palliative, as opposed to supportive, might originate with the cardiac surgeon rather than with the patients or their families. The solution, he suggested, lies in the ability of the cardiac surgeons and intensivists to understand the importance of PC, to view it not as an alternative but as a complement to disease-modifying approaches [24].

In any case, despite the efforts of the WHO and MASCC, there is substantial confusion surrounding the meanings of the two terms [25]. The United States National Cancer Institute lists comfort care, supportive care, and symptom management as synonyms of palliative care [26]. In contrast, other authors regard PC as an intervention used in the most advanced phases of incurable diseases, whereas supportive therapies are those that can be used in the earliest stages of illness [27]. This distinction was adopted by the North American Consensus Conference on Advanced HF [28]. The statement notes that interdisciplinary SC is necessary throughout the entire course of the patient’s HF, right from the diagnosis, dealing, as it does, with the psychosocial aspects of the disease, symptom control, measures for improving the patient’s quality of life and for diminishing their stress and that of their families. The difficulties in defining the prognosis should prompt cardiologists and their patients to agree on a personalized plan of care that includes the definition of the advance directives of the treatment that will be provided in the final stages of life. Therapeutic and supportive interventions in HF patients should not be delivered sequentially but simultaneously [28]. The ESC’s position statement on the use of PC in patients with HF embraces the WHO’s definition of PC, but also includes SC, defined as interventions that are aimed at alleviating the symptoms, complications, and side effects of the treatments and providing support for patients and their families in coping with the disease and the effects of the treatment [29]. In this case as well, there is an overlap, albeit partial, between the meanings of the two terms. No such overlap is to be found in the ESC’s latest position paper on advanced HF, which devotes a paragraph exclusively to PC [5].

That there is some confusion and interchangeability between the two terms is also evident in the work of Lewin et al., who integrated PC specialists into the HF team from the very start, establishing a service referred to as “supportive cardiology” [30]. Similar initial experiences of the integration of PC and HF care in a heart failure supportive care clinic have been described by Laforest et al. in Canada [31], and by members of the Cleveland Clinic [32]. Based on his experience [33] in the field of oncology, Caprio concluded that changing the term used to refer to PC can facilitate its acceptance, and this may well be true for non-cancer disease fields in general.

However, if this type of care continues to be proposed only when patients are in the final stages of their illness, when disease-modifying therapies are being discontinued because they are ineffective or are refused by the patient, the stigma attached to this service will persist. In other words, change at the level of terminology alone is no more than a cosmetic intervention. 

## 4. Prognosis as Another Barrier

It is well known that unlike in cancer patients, in HF cases, it is often difficult to formulate a short- to medium-term prognosis, particularly as HF has an extremely variable clinical course, with alternating phases of acute HF and phases of prolonged relative stability. This is especially true of advanced HF, in which palliative care needs to be implemented as part of the overall treatment program [34].

It is important to stress that the difficulties involved in formulating a short- to mid-term prognosis for a HF Patient [4] are accompanied by substantial differences in the judgments of the various components of the care team [35]. In a study that compared the abilities of internists, cardiologists, and oncologists to predict survival in three illustrative clinical cases, the oncologists made more accurate predictions of survival in a case of advanced lung cancer than cardiologists did in a case of advanced HF (78% vs. 48%) [35].

However, prognosis is still frequently used as a principal tool to decide whether to start PC, even if, in individual patients, the use of prognostic scores derived from clinical trials may often be inadequate [36].

Using prognosis to start PC delays the implementation. 

The “surprise question” (SQ) (“Would I be surprised if this patient died in the next 12 months?”) has been proposed as an effective tool for identifying patients with an advanced chronic disease who need palliative care [37]. It has been incorporated into various algorithms and used in populations of patients with various chronic diseases [38,39,40]. It has also come to be used as a prognostic tool in patients with oncological and non-oncological diseases, but controversial results have emerged in both clinical contexts [41,42,43,44].

The main limitation of the SQ, when applied as prognostic indicator to HF patients, is its low specificity (22%) [42].

Straw et al. [43], in a prospective study of 129 patients with HF, assessed the ability of the SQ to identify those with unfavorable outcomes at 1 year. A negative response to the SQ was significantly correlated with mortality at 1 year. However, the specificity in the cardiologists’ hands was 59%, with a Positive Predictive Value (PPV) of 52%, while the evaluation of HF nurses showed a specificity of 44% and a PPV of 45%. Patel et al. [45], in their editorial comment, stressed the low concordance between the judgments of team members as a limitation of the SQ as a prognostic tool in this context, and highlighted the multidisciplinary nature of the team as a resource for increasing the likelihood of correctly identifying the patients who truly need specialist palliative care [46].

In essence, the SQ has little or no prognostic value, because one HF patient out of two with a negative response will still be alive after one year [45,46].

Palliative or supportive care measures—or whatever else one might call them—should be included in the care plan from the initial stage of the illness and continued thereafter with revisions and adjustments guided by the clinical needs of the patient and their relatives/caregivers. With early integration of PC professionals into the team directly responsible for the patient’s care, the negative connotations surrounding them in the eyes of patients and their families—albeit unjustified—are likely to be less striking, and their acceptance can allow for a more holistic management of the patient’s problems—clinical, psychological, and spiritual. With this approach, prognosis-oriented care can be transformed into patient needs-oriented care [34].

## 5. Lack of PC Specialists

The lack of access to PC programs is often due to a lack of PC specialist availability. It is calculated that the shortage of PC specialists today is a serious problem (the ratio of PC doctors to eligible patients is 1:808) and in the future, it will be worse due also to 30% of physicians burnout and age (> 56 or older) [47].

In another paper [48], Lupu et al., while emphasizing the doubling number of PC physicians in the US between 2010 and 2016, calculated that the need for PC specialists in the next 20 years will not be met. There will be an imbalance between the demand for PC physicians (between 10,640 and 24,000) and the supply (between 8100 and 19,000). 

## 6. A New Model to Early Integrate PC in HF Patients

Attempts to integrate PC into care plans for patients with HF have generally been unsuccessful for several reasons, including the lack of reliable clinical prognostic criteria for initiating PC [49], the absence of shared and validated models [49], and the confusion related to the terminology [25,27,50].

There are two interrelated problems: when to start HF patients on specialist PC and how to integrate the PC specialists into the care team.

In general, HF patients are referred for PC during an advanced stage of their illness, and this may limit its effectiveness [51]. Earlier initiation of palliative intervention allows for a better control of the symptoms, an improved quality of life (for patients and their caregivers), an improved mood, an increased use of joint care planning, and more substantial reductions in healthcare costs [52]. The decision to initiate PC in a patient with advanced HF should be based on the use of the scores of risk for the disease [36], quality of life indices [53], and indicators of decline/deterioration (PPS) [54].

Early integration of a PC specialist into the team caring for a HF patient is recommended by scientific societies, but the recommendation is rarely implemented [31].

**Figure 1:**
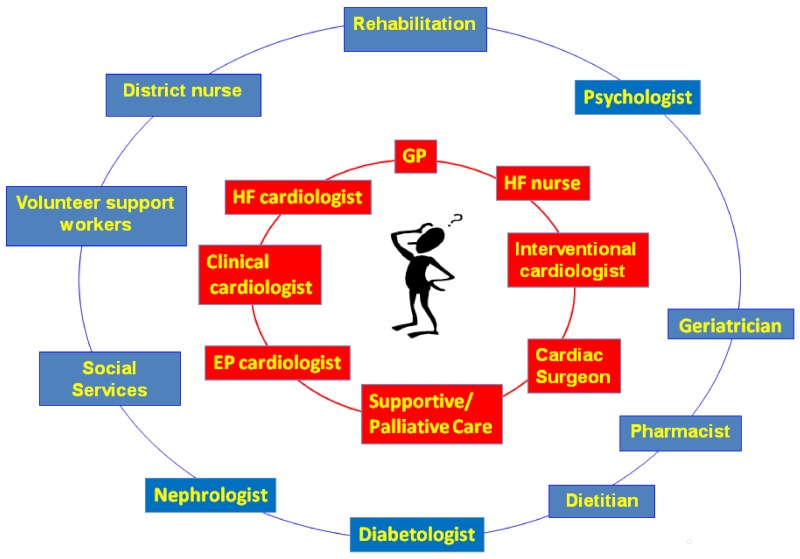
Heart failure clinical network.

The model shown in Figure 1 identifies two concentric rings surrounding the patient: the first ring contains professional figures who, at different times, will need to intervene in the patient’s care and contribute to its planning. The addition to this first ring of PC/SC professionals allows for a more holistic management of the clinical, welfare, psychological, and spiritual problems of the patient and his/her caregivers [34].

**Figure 2:**
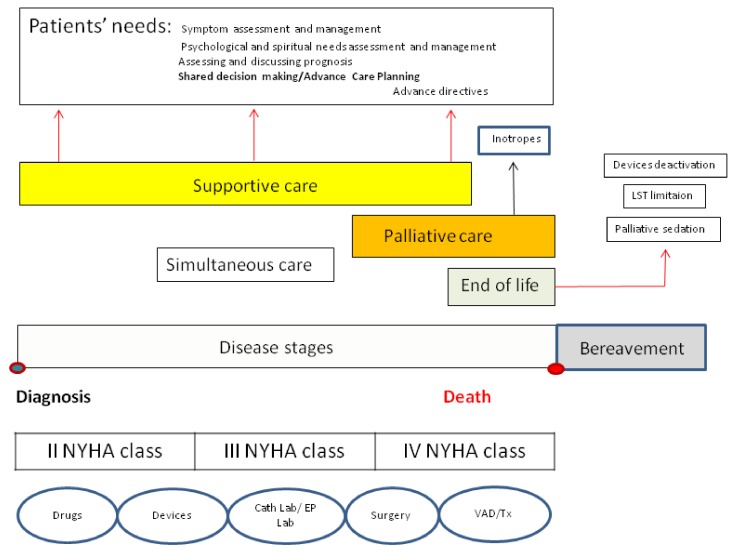
How to integrate patient needs and disease care.

Secondly, the negative stigma attached to PC providers (especially in settings involving chronic non-cancer diseases [30]) could be diminished by rendering these figures members of the medical team caring for the patient. In this manner, parallel care plans could be created from the beginning of the disease course (Figure 2) [27], one for specific disease-modifying therapies, the second devoted to identifying the patient’s needs (diagnosis and therapy of symptoms) and the process of joint care planning (support phase).

VAD: ventricular assist device, Tx: cardiac transplant, EP Lab: electrophysiologic laboratory, Cath lab: catheterization laboratory modified from Hui et al. 27

In subsequent phases, issues related to the implementation of planned treatments will be dealt with, including those destined for the most advanced stages of HF. This point heralds the beginning of the palliative phase of the patient’s care in sensu strict, when interventions such as palliative inotrope therapy are used [5], and the phase of EOL care when the interruption of life-support therapies, the deactivation of implanted cardiac devices, and the use of palliative sedation need to be addressed. 

To improve the effectiveness of this model and to make it applicable in a widespread manner, there is a need for cultural contamination among the different physicians involved.

The PC team could learn the main problems in the clinical course of HF patients, with special attention paid to device management (ICDs, PMs, LVADs) at the end of life.

The cardiologists might improve their attention to symptom evaluation and treatment; moreover, they could start dealing with particularly difficult conversations.

Finally, this cooperation should improve the process of shared decision making in all stages of heart failure.

## 7. Conclusions

The barriers to early implementation of PC in HF patients are still able to make the care insufficient and ineffective.

The knowledge and attitudes of cardiologists in this field is quite low, and this also arises from the assumption that the frequent use of high-tech treatments, even in late HF stages, is beneficial.

The best therapy for all diseases should be built on criteria designed to ensure proportionality of care, which is based on balancing the appropriateness and burdensomeness of treatments and adopting a correct, bioethical approach.

The concept of proportionality is strictly linked with the patient–doctor relationship, which could be improved through a new approach towards HF patients, as described in this review;otherwise, it could be only the rough application of therapeutic techniques.

Cultural changes and the continuous education of physicians are the only solutions to these still unresolved issues.
